# Cilostazol prevents retinal ischemic damage partly via inhibition of tumor necrosis factor-α-induced nuclear factor-kappa B/activator protein-1 signaling pathway

**DOI:** 10.1002/prp2.6

**Published:** 2013-10-01

**Authors:** Fumiya Ishizuka, Masamitsu Shimazawa, Yusuke Egashira, Hiromi Ogishima, Shinsuke Nakamura, Kazuhiro Tsuruma, Hideaki Hara

**Affiliations:** 1 Molecular Pharmacology, Department of Biofunctional Evaluation, Gifu Pharmaceutical University1-25-4 Daigaku-nishi, Gifu, 501-1196, Japan; 2 Department of Neurosurgery, Gifu University Graduate School of MedicineGifu, Japan

**Keywords:** Activator protein-1, cilostazol, inflammation, nuclear factor-kappa B, ocular ischemic syndrome, phosphodiesterase III inhibitor, retinal microvascular endothelial cells, tight junction proteins, tumor necrosis factor-α

## Abstract

Cilostazol is a specific inhibitor of phosphodiesterase III and is widely used to treat ischemic symptoms of peripheral vascular disease. We evaluated the protective effects of cilostazol in a murine model of ocular ischemic syndrome in which retinal ischemia was induced by 5-h unilateral ligation of both the pterygopalatine artery (PPA) and the external carotid artery (ECA) in anesthetized mice. The effects of cilostazol (30 mg/kg, p.o.) on ischemia/reperfusion (I/R)-induced retinal damage were examined by histological, retinal vascular permeability, and electrophysiological analyses. Using immunoblotting, the protective mechanism for cilostazol was evaluated by examining antiinflammatory effects of cilostazol on the expression of tumor necrosis factors-α (TNF-α) and tight junction proteins (ZO-1 and claudin-5), and the phosphorylations of nuclear factor-kappa B (NF-κB) and c-Jun. The histological analysis revealed that I/R decreased the cell number in the ganglion cell layer (GCL) and the thicknesses of the inner plexiform layer (IPL) and inner nuclear layer (INL), and that cilostazol attenuated these decreases. Additionally, cilostazol prevented the hyperpermeability of blood vessels. Electroretinogram (ERG) measurements revealed that cilostazol prevented the I/R-induced reductions in a-, b-, and oscillatory potential (OP) wave amplitudes seen at 5 days after I/R. Cilostazol inhibited the increased expression of TNF-α and the phosphorylation levels of NF-κB and c-Jun in the retina after I/R. In addition, cilostazol prevented TNF-α-induced reduction of ZO-1 and claudin-5 expression in human retinal microvascular endothelial cells (HRMECs). These findings indicate that cilostazol may prevent I/R-induced retinal damage partly through inhibition of TNF-α-induced NF-κB/AP-1 signaling pathway.

## Introduction

Ocular ischemic syndrome (OIS) is a condition that is caused by ocular hypoperfusion due to stenosis or occlusion of the common or internal carotid arteries. Severe carotid artery stenoses or occlusions related to atherosclerosis are the major causes of OIS. Visual loss is the most frequent symptom encountered in OIS and it is observed in more than 90% of affected persons (Brown and Magargal 1988), but retinal hemorrhage, macular edema, and narrowed retinal arteries are also frequently found in OIS patients (Terelak-Borys et al. 2012). Thus far, a therapeutic agent has not been developed for OIS. Recently, we reported a new murine OIS model in which both the pterygopalatine artery (PPA) and the external carotid artery (ECA) were ligated (Ogishima et al. 2011). This animal model is useful both for the clarification of the pathologic mechanisms underlying OIS and for the evaluation of neuroprotective drugs that might be used to target that syndrome.

Cilostazol is an antiplatelet drug and vasodepressor. The main pharmacological effect of cilostazol is an increase in the level of intracellular cyclic AMP (cAMP) through inhibition of phosphodiesterase III (Tanaka et al. 1989). Cilostazol has been approved for use as a vasodilating antiplatelet drug for the treatment of ischemic symptoms in chronic peripheral arterial obstruction or intermittent claudication, and for secondary prevention of cerebral infarction, and has been examined in ischemic stroke prevention clinical trials (Matsumoto 2005; Lee et al. 2008; Shinohara et al. 2010). In the rat retina, it was shown that cilostazol is protective during retinal ischemic injury through suppression of the interaction of leukocytes and endothelial cells (Iwama et al. 2007), and enhances the survival of axotomized retinal ganglion cells (RGCs) through activation of the protein kinase A (PKA) pathway (Kashimoto et al. 2008). Moreover, the results of a recent randomized trial (Cilostazol for Prevention of Secondary Stroke [CSPS II]) indicated that cilostazol is more effective than aspirin in the secondary prevention of all types of stroke in patients and, in particular, in preventing secondary attacks of hemorrhagic stroke (Shinohara et al. 2010). In nonclinical studies, we also showed that cilostazol protects against hemorrhagic transformation in mouse transient focal cerebral ischemia (Nonaka et al. 2009b; Shimazawa and Hara 2011) and that it prevents hemorrhagic transformation induced by focal cerebral ischemia in mice treated with tissue plasminogen activator (tPA) (Ishiguro et al. 2010).

From these findings, we hypothesized that cilostazol will be neuroprotective against retinal ischemic injury in a model of OIS, and that it might inhibit the retinal vascular hyperpermeability induced by ischemia/reperfusion (I/R). To test this hypothesis and explore the underlying mechanisms of cilostazol in retinal ischemia, we utilized histological and electrophysiological analyses, and assessed retinal vascular permeability.

## Materials and Methods

### Animals

Male ddY mice (closed colony albino mice; Japan SLC, Hamamatsu, Japan), aged 8–9 weeks, were used in this study. They were kept under controlled lighting conditions (12 h:12 h light/dark). All experiments were performed in accordance with the Association for Research in Vision and Ophthalmology Statement for the Use of Animals in Ophthalmic and Vision Research, and they were approved and monitored by the Institutional Animal Care and Use Committee of Gifu Pharmaceutical University.

### Retinal ischemia model in mice

Mice were initially anesthetized by means of 2.5–3.0% isoflurane and maintained throughout the surgery with 1.5% isoflurane (both in 70% N_2_O/30% O_2_) delivered through an animal anesthesia machine (Soft Lander; Sin-ei Industry Co. Ltd., Saitama, Japan). Body temperature was maintained between 37.0°C and 37.5°C with the aid of a temperature control system (NS-TC 10; Neuroscience Inc., Tokyo, Japan). Retinal ischemia was induced by PPA and ECA ligations, as described in our previous reports (Ogishima et al. 2011; Ishizuka et al. 2013). Briefly, after a midline skin incision, the left common carotid artery was exposed, and the ECA was ligated. The internal carotid artery and its first branch were dissected, and the PPA was ligated. Ischemia was maintained for 5 h, and then the ligatures were removed. All the above procedures were performed while the animal was under anesthesia.

### Treatment with cilostazol

For this study, cilostazol was kindly gifted by Otsuka Pharmaceutical Co. Ltd. (Tokushima, Japan). It was suspended in distilled water containing 0.5% sodium carboxymethyl cellulose (CMC) immediately before use. Mice were treated orally either with cilostazol at a dose of 3 or 30 mg/kg or with an identical volume (see below) of CMC. Each mouse received one of those doses at just after reperfusion and then once a day for the next 4 days. The dosage volume was adjusted to be 10 mL/kg body weight.

### Histology

At 5 days after ligation, mice were anesthetized by intraperitoneal injection of pentobarbital sodium (80 mg/kg; Nacalai Tesque, Kyoto, Japan), and then euthanized. Left eyes were enucleated and kept immersed for at least 24 h at 4°C in a fixative solution containing 4% paraformaldehyde. Nine paraffin-embedded sections (thickness, 5 μm) were cut through the optic disc parallel with the maximum circle of the eyeball. Following extraction of the eyeball, the top of the dorsal point was identified (using the optic axis as the landmark) and stained with hematoxylin and eosin. The damage induced by retinal ischemia was evaluated as described below, with three sections from each eye being used for the morphometric analysis. On photographs of light microscope images, the cell counts in the ganglion cell layer (GCL) between 375 and 625 μm from the optic disc (nasal and temporal portions), and the thickness of the inner plexiform layer (IPL) and that of the inner nuclear layer (INL) were measured at two points per section on the photographs. Those values were then averaged. Data from three sections were averaged for each eye and these values were entered into the analysis of the cell count in the GCL and the thickness of the IPL and of the INL.

### Permeability of retinal blood vessels

The permeability of mouse retinal vessels was measured by the modification of the previous method (Koto et al. 2007). Briefly, each mouse was intravenously injected with 0.5 mL of PBS containing 100 mg/mL Hoechst 33,342 (molecular mass, 616 Da; Sigma-Aldrich, St. Louis, MO) and 20 mg/mL FITC-dextran (molecular mass, 2000 kDa; Sigma-Aldrich). The isolated retinas were flat mounted and observed using a confocal microscopy (FluoView FV10; Olympus, Tokyo, Japan). Moreover, to quantify retinal permeability using Metamorph (Universal Imaging Corp., Downingtown, PA), fluorescent images were photographed (200×, 0.144 mm^2^) using an epifluorescence microscope (BX50; Olympus) fitted with a CCD camera (DP30VW; Olympus). Areas of Hoechst 33,342 that did not merge with FITC-dextran at a distance of 1 mm from the optic disc were evaluated on the photographs in a masked fashion by a single observer (F.I.). Data from the four parts of each eye were used (total area, 0.576 mm^2^). The following equation was used to calculate retinal permeability rate:

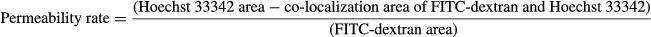


### Electroretinogram recording

Five days after the I/R, electroretinogram (ERG) recordings were performed as explained in our previous report (Imai et al. 2010). Scotopic ERG records were used to evaluate retinal function in mice that had been kept in a completely dark room for 24 h. They were anesthetized intraperitoneally with a mixture of ketamine (120 mg/kg; Daiichi-Sankyo, Tokyo, Japan) and xylazine (6 mg/kg; Bayer Health Care, Tokyo, Japan), and their pupils were dilated with 1% tropicamide and 2.5% phenylephrine (Santen Pharmaceutical Co., Ltd., Osaka, Japan). Flash ERG was recorded in the left eye of each dark-adapted mouse by placing a golden-ring electrode (Mayo, Aichi, Japan) in contact with the cornea and a reference electrode (Nihon Kohden, Tokyo, Japan) through the tongue. A neutral electrode (Nihon Kohden) was inserted subcutaneously near the tail. All procedures were performed in dim red light, and the mice were kept warm throughout the procedure. The amplitude of the a-wave was measured from the baseline to the highest a-wave peak, and the b-wave was measured from the highest a-wave peak to the highest b-wave peak. To analyze the oscillatory potentials (OPs), the OP amplitudes were measured in the time between the a- and b-wave peaks; the factors were OP number (OP2, OP3, OP4, and OP5), and flash intensity (0.98 [log cds/m^2^]). In accordance with a previous report (Akula et al. 2007), the first OP waves were not analyzed. OPs were isolated by the band pass filter, and OPs amplitudes were measured by using ERG with all frequencies (0.3–500 Hz). The four OPs have been enumerated on each trace.

### Western blot analysis

Mice were euthanized by cervical dislocation, their eyeballs rapidly removed, and the retinas carefully separated from the eyeballs and quickly frozen in dry ice. For protein extraction, the tissue was homogenized in cell lysis buffer using a Physcotron homogenizer (Microtec Co. Ltd., Chiba, Japan). The lysate was centrifuged at 12000 *g* for 20 min, and the supernatant was used for this study. The protein concentrations were measured by comparison with a known concentration of bovine serum albumin using a bicinchoninic acid protein assay kit (Pierce Chemical, Rockford, IL). A mixture of equal parts of an aliquot of protein and sample buffer with 10% 2-mercaptoethanol was subjected to 15% sodium dodecyl sulfate-polyacrylamide gel electrophoresis. The separated protein was then transferred onto a polyvinylidene difluoride membrane (Immobilon-P; Millipore Corporation, Billerica, MA). Transfers were blocked for 1 h at room temperature with 5% Block One-P (Nacalai Tesque Inc., Kyoto, Japan) in 10 mmol/L Tris-buffered saline with 0.05% Tween 20, then incubated overnight at 4°C with the primary antibody. For immunoblotting, the following primary antibodies were used: rabbit anti-phosphorylated-c-Jun monoclonal antibody (Cell Signaling, Danvers, MA) (1:1000), rabbit anti-phosphorylated-nuclear factor-kappa B (NF-κB) monoclonal antibody (#3303, Cell Signaling) (1:1000), rabbit anti-c-Jun monoclonal antibody (Cell Signaling) (1:1000), rabbit anti-NF-κB polyclonal antibody (Cell Signaling) (1:1000), rabbit anti-tumor necrosis factors-α (TNF-α) polyclonal antibody (Cell Signaling) (1:100), rabbit anti-zonula occludens 1 (ZO-1) polyclonal antibody (Santa Cruz Biotechnology, Santa Cruz, CA) (1:200), rabbit anti-claudin-5 polyclonal antibody (Santa Cruz) (1:200), and mouse anti-β-actin monoclonal antibody (Sigma-Aldrich) (1:5000). Horseradish peroxidase-conjugated goat anti-rabbit or anti-mouse antibody (1:2000) was used as a secondary antibody. The immunoreactive bands were visualized using Immuno Star® LD (Wako Pure Chemical, Osaka, Japan), then measured using LAS-4000 Mini (Fuji Film Co., Ltd., Tokyo, Japan).

### Human retinal microvascular endothelial cell culture and treatment

Primary human retinal microvascular endothelial cells (HRMECs) were obtained from DS Pharma Biomedical (Osaka, Japan). They were seeded at a density of 5 × 10^4^ cells per well on 12-well culture plates, and incubated in CS-C medium and culture boost (growth factors) (Cell Systems, Kirkland, WA) at 37°C in 5% CO_2_ until they reached 70% confluence. To investigate the effects of cilostazol on the TNF-α induced inflammation model, we used methods described previously (Guo et al. 2010). Briefly, the culture medium was removed and cells were washed twice with PBS. The cells were then treated with varying concentrations of cilostazol and 10 ng/mL of TNF-α (Recombinant Human TNF-α; R&D systems Inc, Minneapolis, MN) for 20 h. Following these treatments, cell lysates were collected, and Western blot analysis was performed in order to assess the expression levels of ZO-1 and claudin-5, using the immunostaining protocol described above.

### Statistical analysis

Data are presented as the means ± SEM. Statistical comparisons were made using a two-tailed Student's *t*-test or Dunnett's test by means of STATVIEW version 5.0 (SAS Institute, Cary, NC). If the *P*-value is less than 0.05, the difference was considered statistically significant.

## Results

### Cilostazol protected against the histological damage in the mouse retina induced by ischemia/reperfusion

**Figure 1:**
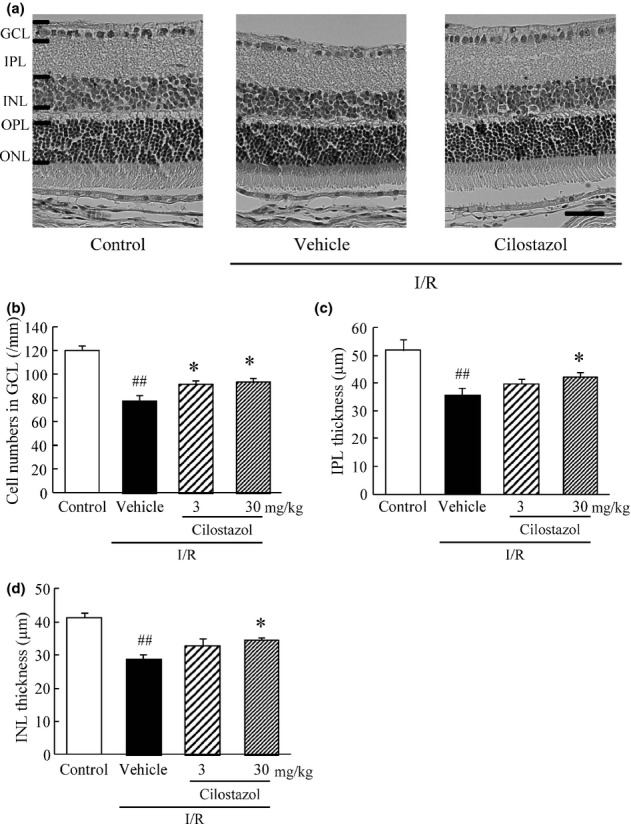
Effects of cilostazol on ischemia/reperfusion-induced retinal morphological damage. (A) Representative photographs of hematoxylin and eosin staining of retinal sections showing retinas treated with vehicle, ischemia/reperfusion (I/R) plus vehicle, or I/R plus cilostazol (30 mg/kg). Scale bar represents 20 μm (B–D) Quantitative analysis of the cell number in ganglion cell layer (GCL) (B), and of the thickness of (C) inner plexiform layer (IPL) and of (D) inner nuclear layer (INL), all at 5 days after I/R. Data are shown as means ± SEM (*n* = 8–10). ^##^*P* < 0.01 versus control; *P < 0.05 versus I/R plus vehicle (vehicle).

Figure 1A shows representative retinal images at 5 days after ischemia/reperfusion. I/R caused decreases in the cell number in the GCL and thinning of the IPL and INL in the mouse retina at 5 days after ischemia compared to control (no I/R) retinas. The groups treated with cilostazol (3 and 30 mg/kg) significantly reduced the decreases in cell number in GCL induced by ischemia (Fig. 1B). Furthermore, cilostazol (30 mg/kg) significantly attenuated the decreased thickness of the IPL and INL induced by ischemia (Fig. 1C and D).

### Cilostazol suppressed the increase of retinal permeability in the murine retina induced by ischemia/reperfusion

**Figure 2:**
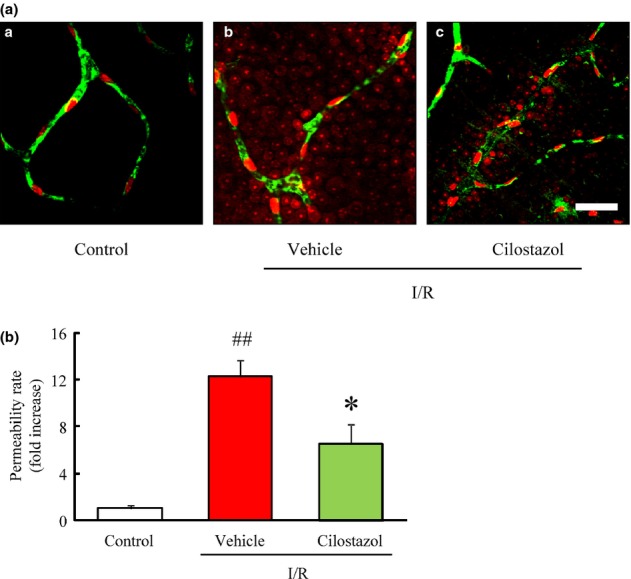
Effects of cilostazol on the ischemia/reperfusion-induced increase in retinal vascular permeability in mice. (A-a) Confocal fluorescence micrographs of vehicle-treated, (b) ischemia/reperfusion (I/R) plus vehicle-treated, (c) I/R plus cilostazol-treated (30 mg/kg) mice retina. Fluorescein isothiocyanate (FITC)-dextran is green and confined to blood vessels, whereas Hoechst 33,342 is red and present outside of the blood vessels. Scale bar represents 40 μm. (B) Quantitative analysis of retinal permeability at 5 days after I/R. Data are presented at means ± SEM (*n* = 6–8). ^##^*P* < 0.01 versus control; *P < 0.05 versus I/R plus vehicle (vehicle).

The permeability of retinal vessels was evaluated by tracer experiments using FITC-dextran (2000 kDa) and Hoechst 33342 (616 Da). The injected FITC-dextran, a tracer molecule of larger size, was detected in the vascular lumen with minimal leakage from blood vessels (Fig. 2, green). The extravasation of the Hoechst 33342 dye appeared in the nuclear staining of surrounding retinal glial and neural cells and the degree of staining was enhanced in the vehicle-treated I/R group compared to the control group (Fig. 2, red). Quantitative analysis showed that the retinal permeability rate was significantly greater in the vehicle-treated I/R group compared to the control group, and that treatment with cilostazol (30 mg/kg) significantly suppressed the increase of retinal permeability rate after I/R compared to vehicle treatment (Fig. 2).

### Cilostazol inhibited the functional damage in the murine retina induced by ischemia/reperfusion

**Figure 3:**
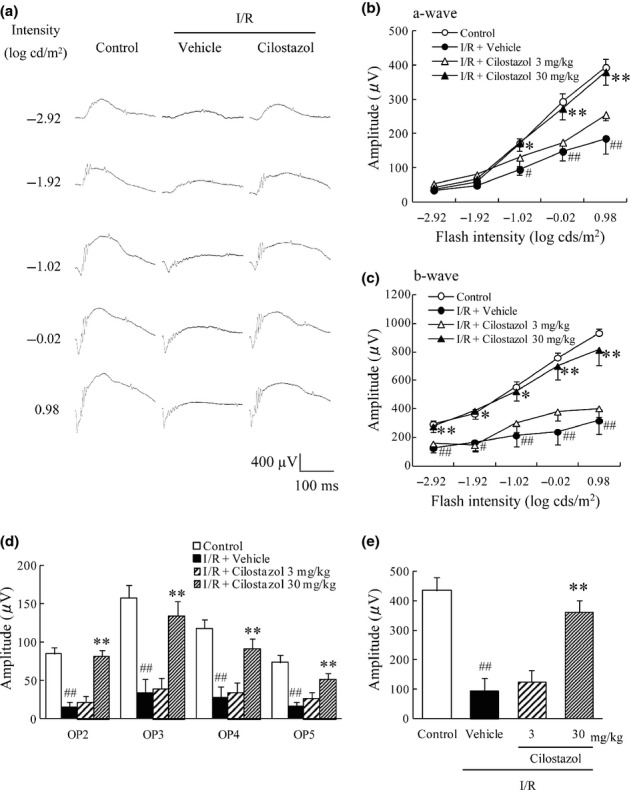
Effects of cilostazol on ischemia/reperfusion-induced retinal functional damage. The effects of cilostazol were evaluated by recording the electroretinogram (ERG) at 5 days after ischemia/reperfusion (I/R). (A) Representative ERG recordings from retinas treated with vehicle, I/R plus vehicle or I/R plus cilostazol (30 mg/kg). (B, C) Intensity-response functions for dark-adapted (B) a-wave and (C) b-wave amplitudes. The cilostazol-treated group showed significant preservation of both a- and b-wave amplitudes compared with the vehicle-treated group. (D) The averaged oscillatory potential (OP) amplitudes in response to a light flash of 0.98 log cd/m^2^. (E) The sum of amplitudes of the four OPs. Data are shown as means ± SEM (*n* = 8–10). ^#^*P* < 0.05; ^##^*P* < 0.01 versus control; *P < 0.05; **P < 0.01 versus I/R plus vehicle (vehicle).

The effects of cilostazol on I/R-induced retinal dysfunction were examined by electrophysiological analysis. In the control (no I/R) group, both the a-wave and b-wave amplitudes increased in a light intensity-dependent manner (Fig. 3B and C). Each of these amplitudes was significantly reduced in the vehicle-treated I/R group compared to the control group at 5 days after I/R. Treatment with cilostazol at 30 mg/kg, but not 3 mg/kg, rescued retina function after I/R, showing significant reversal of each dysfunction compared with the vehicle-treated I/R group (Fig. 3B and C). The OP amplitudes were measured under scotopic conditions in response to a flash (0.98 log cd/m^2^). The mean amplitude of isolated OPs (Fig. 3D) and the sum of all isolated OPs (Fig. 3E) were severely reduced after I/R. Cilostazol (30 mg/kg) significantly inhibited the reduction in the mean amplitude of these OPs induced by retinal I/R, but cilostazol at 3 mg/kg did not.

### Cilostazol prevented the ischemia/reperfusion-induced activation of proinflammatory mediators in the murine retina

**Figure 4:**
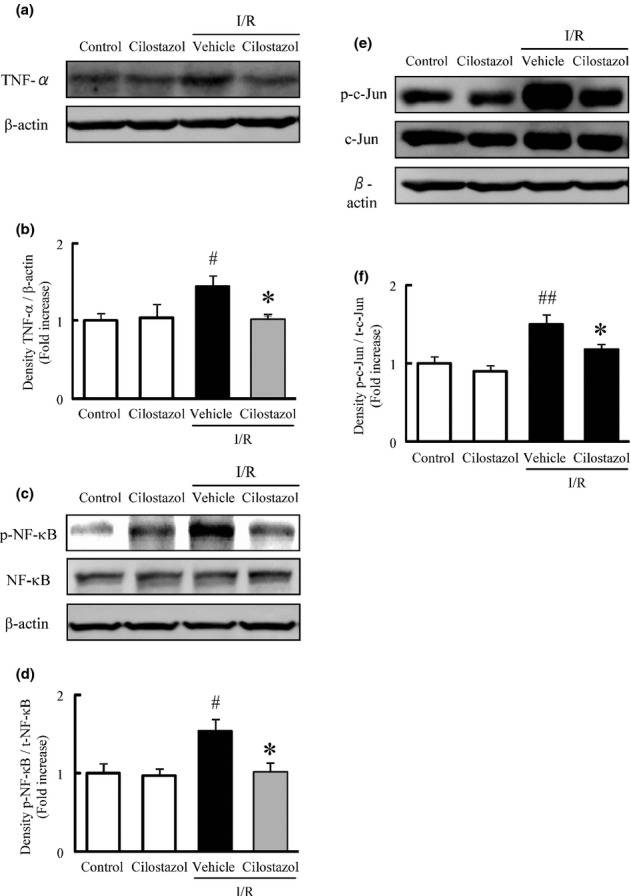
Effects of cilostazol on ischemia/reperfusion-induced activation of proinflammatory mediators in the murine retina. (A, C, E) Representative band images showing (A) tumor necrosis factor-α (TNF-α), (C) nuclear factor-κB (NF-κB), and (E) c-Jun in retinas treated with vehicle, cilostazol, ischemia/reperfusion (I/R) plus vehicle, or I/R plus cilostazol. (B, D, F) Quantitative analysis of band densities for (B) TNF-α, (D) NF-κB, and (F) c-Jun. Data are shown as means ± SEM (*n* = 5 or 6). ^#^*P* < 0.05; ^##^*P* < 0.01 versus control; *P < 0.05 versus I/R plus vehicle (vehicle).

The effects of cilostazol on the I/R-induced activation of proinflammatory factors were evaluated by measuring the expression level of TNF-α and the phosphorylations of c-Jun and NF-κB through Western blot analysis. The expression level of soluble TNF-α (17 kDa) in the murine retina was significantly increased 24 h after the onset of ischemia in comparison to the control group. Treatment with cilostazol at 30 mg/kg significantly suppressed the I/R-induced increase in expression of TNF-α (Fig. 4A and B). Additionally, the ratio of phosphorylated NF-κB to total NF-κB was significantly increased 24 h after the onset of ischemia when compared to the control group (Fig. 4C and D), while the ratio of phosphorylated c-Jun to total c-Jun was significantly increased 12 h after the onset of ischemia compared to the control group (Fig. 4E and F). Cilostazol (30 mg/kg) significantly reduced the I/R-induced increase in phosphorylation of both NF-κB and c-Jun.

### Cilostazol prevented the reduction of tight junction proteins in TNF-α-treated HRMECs in vitro

**Figure 5:**
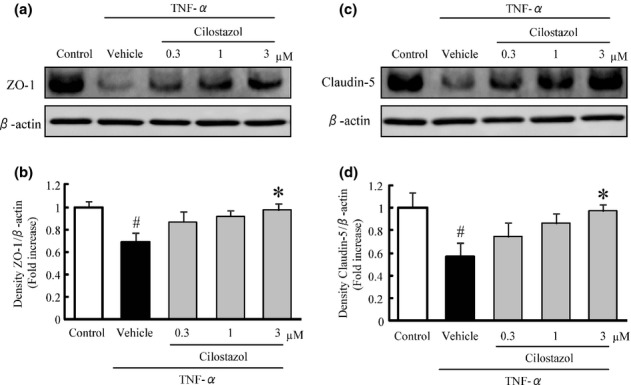
Effects of cilostazol on tumor necrosis factor-α (TNF-α)-induced disruption of tight junction proteins in the human retinal microvascular endothelial cells (HRMECs). (A, C) Representative band images for (A) zonula occludens 1 (ZO-1) and (C) claudin-5 in HRMECs. (B, D) Quantitative analysis of the band densities for ZO-1 (B) and claudin-5 (D). Data are shown as means ± SEM (*n* = 3 or 4). ^#^*P* < 0.05 versus control; *P < 0.05 versus vehicle.

In this study, HRMECs treated with TNF-α were used as an in vitro inflammatory model of retinal endothelial cells. To investigate the effect of cilostazol on the disruption of tight junctions induced by TNF-α, we evaluated the expression levels of ZO-1 and claudin-5 in HRMEC after treatment with TNF-α in the presence or absence of cilostazol. After 20 h of exposure to TNF-α (10 ng/mL), the protein expression levels of ZO-1 (Fig. 5A and B) and claudin-5 (Fig. 5C and D) were significantly decreased compared to untreated control cells. The decreased expression of ZO-1 and claudin-5 was reversed by cilostazol in a concentration-dependent manner, with significant reversal observed at 3 μmol/L.

## Discussion

In this study, oral administration of cilostazol protected against I/R-induced retinal cell death and retinal dysfunction, and inhibited the I/R-induced increase of retinal vascular permeability. Cilostazol also reduced the I/R-induced activation of proinflammatory mediators in the murine retina and inhibited the TNF-α-induced disruption of tight junctions in HRMEC.

The present result, that cilostazol prevented the retinal damage after I/R, is in agreement with previous reports about optic nerve injury and cerebral ischemic injury induced by ligation of the optic sheath and middle cerebral artery occlusion, respectively (Iwama et al. 2007; Nonaka et al. 2009a). These findings suggest that cilostazol has a protective effect against the retinal cell death induced by I/R.

An increase in retinal vascular permeability was found in retinas exposed to retinal artery occlusion (Kaur et al. 2008), high intraocular pressure (Abcouwer et al. 2010), and oxygen-induced retinopathy (OIR)-induced retinal ischemia (Zhang et al. 2005). Cilostazol has been reported to attenuate the hyperpermeability of endothelial cells in hypoxia/reperfusion (Torii et al. 2007), and to have a protective effect against oxygen-glucose deprivation stress in endothelial cells (Ishiguro et al. 2011). In this study, an increase in retinal vascular permeability was caused by ligation of the PPA and ECA in an experimental OIS model in mice, and it was suppressed by the administration of cilostazol. This result supports the idea that cilostazol is protective against the retinal endothelial cell damage. On the other hand, cilostazol has a vasodilating effect on retinal arteries and increases ocular blood flow (Hotta et al. 1998; Suzuki et al. 1998). Therefore, we could not exclude the possibility of ocular circulation improvement on the neuroprotective effect of cilostazol against I/R injury in vivo.

According to previous reports, both the a-wave and the b-wave of the ERG are reduced in amplitude after retinal ischemia (Grozdanic et al. 2003; Ogishima et al. 2011), which is in keeping with our results. ERG recording is used as an objective way of evaluating the functional status of the inner and outer retinas; the a-wave reflects the function of the photoreceptors and the b-wave reflects the functions of bipolar cells and Müller cells. In this study, the amplitudes of both the a- and b-waves were reduced following I/R, as were the amplitudes of OPs. Cilostazol largely reversed all of these reductions. Although the origin of OPs has not been definitively determined, OPs are thought to originate from feedback neural pathways in the inner retina, especially around IPL, mainly from amacrine cells, although ganglion cells and bipolar cells may contribute to some aspects of the OPs (Heynen et al. 1985; Wachtmeister 1998; Rangaswamy et al. 2006). Recently, it was reported that the amplitudes of OPs were markedly decreased in the region where retinal permeability was increased in patients with diabetes and clinically significant macular edema (Greenstein et al. 2000). Similarly, the attenuation of amplitudes of OPs might affect retinal hyperpermeability in the chronic phase retina of OIR mice (Nakamura et al. 2012). Therefore, the inhibition by cilostazol of the abnormalities of OPs might result from suppression of the increase of retinal vascular permeability. The above findings indicate that cilostazol can attenuate the photoreceptor and inner retinal dysfunctions induced by I/R.

Inflammation occurs in the retina in I/R injuries (Zhang et al. 2009). TNF-α is a proinflammatory cytokine, and can lead to apoptosis (Berger et al. 2008). Responses to TNF-a have been reported to be diverse and mediated by the activation of extrinsic apoptotic pathways such as NF-κB, MAPK, and PI3K/Akt. In addition, TNF-α causes hyperpermeability of endothelial cells (Abe et al. 1990). Cilostazol inhibits the I/R-induced increase of TNF-α in the rat brain, and the inhibition might be closely related to increased cyclic AMP levels (Choi et al. 2002; Lee and Eun 2012). In this study, a soluble form of TNF-α was increased after retinal I/R and cilostazol inhibited this increase. Taken together, cilostazol might inhibit the retinal cell death and the increase in vascular permeability induced by retinal I/R by suppressing the increase of TNF-α expression.

**Figure 6:**
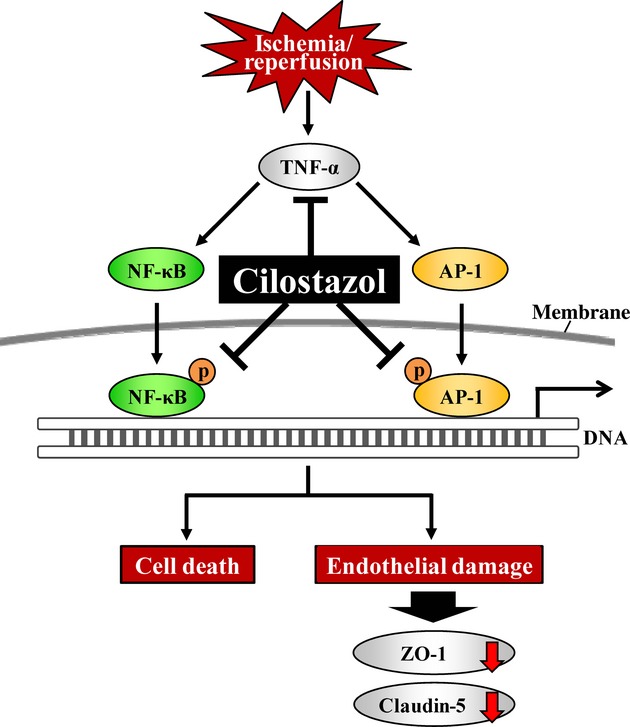
Putative mechanism for protective effects of cilostazol against retinal damage induced by ischemia/reperfusion. The schematic demonstrates the putative mechanism of cilostazol interference in ischemia/reperfusion (I/R)-induced disruptions of retinal function including the production of tumor necrosis factor-α (TNF-α) and the activation of nuclear factor-κB (NF-κB) and activator protein 1 (AP-1). These effects of cilostazol lead to protective effects on retinal cell death and endothelial damage, resulting in decreasing expression levels of ZO-1 and claudin-5.

Changes in retinal vascular permeability might result from alterations of the tight junction complex. Tight junctions are composed of a combination of many proteins, and ZO-1 and claudin-5 are essential for the formation and organization of the tight junction complex assembly (Nitta et al. 2003; Umeda et al. 2006; Katsuno et al. 2008). Previously, it was demonstrated that cilostazol increased the intracellular cyclic AMP level in rat brain capillary endothelial cells (Horai et al. 2013), and cAMP analogs have been shown to enhance the barrier function of tight junction in brain capillary endothelial cells (Stelzner et al. 1989). Moreover, cilostazol prevented the decrease in claudin-5 induced by tPA-associated hemorrhagic transformation (Ishiguro et al. 2010). On the other hand, the inhibition of TNF-α with etanercept, a soluble TNF-α receptor antagonist, inhibited the breakdown of the blood-retinal barrier (BRB) in the diabetic rat retina (Saishin et al. 2003). TNF-α increased the bovine retinal endothelial cell permeability, and decreased the expression of the tight junction proteins, ZO-1 and claudin-5 (Aveleira et al. 2010). These reports indicate that TNF-α contributes to vascular permeability in the retina after ischemia. In this study, the expression of tight junction proteins ZO-1 and claudin-5 in HRMEC treated with TNF-α was decreased, and cilostazol reversed these decreases in a concentration-dependent manner. According to previous reports, TNF-α-induced retinal endothelial cell hyperpermeability is mediated by NF-κB (Aveleira et al. 2010), and JNK signaling plays an important role in the regulation of tight junction proteins and BBB integrity (Chen et al. 2012). Cilostazol has been shown to inhibit the lipopolysaccharide (LPS)-induced NF-κB p65 nuclear translocation and phosphorylation of JNK (Jung et al. 2010; Park et al. 2013). In this study, the phosphorylation of NF-κB and c-Jun, a protein of the AP-1 family phosphorylated by JNK, was inhibited by the administration of cilostazol. We have previously reported that cilostazol and a cAMP analogue both inhibit the cell damage after oxygen-glucose deprivation in cultured human brain microvascular endothelial cells, and an inhibitor of PKA eliminates the protective effect of cilostazol (Ishiguro et al. 2011). This report suggests that cilostazol has direct protective effects on endothelial cells, and its effects are mediated by the activation of cAMP-PKA pathway accompanying its phosphodiesterase III inhibition. Furthermore, Yoshida et al. (2012) investigated the maximum drug concentration (*C*_max_) in the blood plasma after the treatment with cilostazol (30 mg/kg, p.o.) in rats. The *C*_max_ of cilostazol was 2.08 ± 0.28 μmol/L at 3.5 h after the oral treatment, and all findings were consistent with the present in vitro and in vivo results. These results suggest that cilostazol might protect against retinal vascular leakage by inhibiting the production of TNF-α and the activation of NF-κB and MAPK in association with cAMP activity (Fig. 6).

In conclusion, cilostazol may prevent I/R-induced retinal damage partly through inhibition of TNF-α-induced NF-κB/AP-1 signaling pathway. These results indicate that cilostazol has potential as a therapeutic agent for degenerative diseases of the retina, including ocular ischemic syndromes.

## Abbreviations

CMC: carboxymethyl cellulose
CSPS II: Cilostazol for Prevention of Secondary Stroke
ECA: external carotid artery
ERG: electroretinogram
GCL: ganglion cell layer
HRMECs: human retinal microvascular endothelial cells
I/R: ischemia/reperfusion
INL: inner nuclear layer
IPL: inner plexiform layer
OIR: oxygen-induced retinopathy
OIS: ocular ischemic syndrome
ONL: outer nuclear layer
OPL: outer plexiform layer
OPs: oscillatory potentials
PPA: pterygopalatine artery
tPA: tissue plasminogen activator
TUNEL: terminal deoxyribonucleotidyl transferase-mediated dUTP nick-end labeling
ZO: zonula occludens 1
